# Social Media Mining for an Analysis of Nutrition and Dietary Health in Taiwan

**DOI:** 10.3390/nu13061778

**Published:** 2021-05-23

**Authors:** Yu-Lun Hsieh, Yung-Chun Chang, Wei-Yu Chang

**Affiliations:** 1 Institute of Information Science, Academia Sinica, Taipei 115201, Taiwan; morpheus.h@gmail.com; 2 Graduate Institute of Data Science, Taipei Medical University, Taipei 106339, Taiwan; wies2375@gmail.com; 3 Clinical Big Data Research Center, Taipei Medical University Hospital, Taipei 106339, Taiwan

**Keywords:** data mining, social media, Chinese recipes, machine learning, information extraction, Taiwan

## Abstract

Dining is an essential part of human life. In order to pursue a healthier self, more and more people enjoy homemade cuisines. Consequently, the amount of recipe websites has increased significantly. These online recipes represent different cultures and cooking methods from various regions, and provide important indications on nutritional content. In recent years, the development of data science made data mining a popular research area. However, only a few researches in Taiwan have applied data mining in the studies of recipes and nutrients. Therefore, this work aims at utilizing machine learning models to discover health-related insights from recipes on social media. First, we collected over 15,000 Chinese recipes from the largest recipe website in Taiwan to build a recipe database. We then extracted information from this dataset through natural language processing methodologies so as to better understand the characteristics of various cuisines and ingredients. Thus, we can establish a classification model for the automatic categorization of recipes. We further performed cluster analysis for grouping nutrients to recognize the nutritional differences for each cluster and each cuisine type. The results showed that using the support vector machine (SVM) model can successfully classify recipes with an average F-score of 82%. We also analyzed the nutritional value of different cuisine categories and the possible health effects they may bring to the consumers. Our methods and findings can assist future work on extracting essential nutritional information from recipes and promoting healthier diets.

## 1. Introduction

Under the economic prosperity and the rapid development of science and technology, the social pattern has changed dramatically. For example, the rise of social network has greatly changed the culinary culture. The choice of eating or cooking has become a hot topic, as well as the social influence of dietary choices [1]. The immediacy and convenience of the Internet have made sharing both food photos and cooking methods easier. The popularity of food search applications and food sharing websites is also increasing [2]. Researchers have also noticed the connection between recipes and diet habits under the effect of social networks [3].

The choice of recipes reflects one’s preference on ingredients and diet habits, which in turn has a strong correlation with diseases, including incidence rate of cancer [4], the death rate [5], cardiovascular disease [6], and metabolic related diseases and obesity [7]. Moreover, a 2014 study in Brazil [8] examined the association between adult eating habits and metabolic syndrome from a total of 1112 cases. The results showed that the higher intake of fat-containing and sugary foods increased the risk of metabolic syndrome. Other researches [9,10,11] indicated that the current human diet is mostly refined cereals, excessive saturated fatty acids, red meat, processed meats, refined sugars, and fewer fruits, vegetables, whole grains, dietary fiber, plant protein, and nuts, which cause insulin resistance, inflammatory reactions that lead to an increase in the prevalence of chronic diseases, such as obesity and diabetes. In order to promote personal health and provide users with appropriate recipes and ingredients, information systems have been proposed to recommend better diets for individuals [12,13]. In addition, more attention was paid on the linkage between recipes and diseases. In 2011, a study published a mobile app that can find the nutrients needed by users through dietary questions raised by users and recommend suitable recipes. If the user enters “How to treat hemorrhoids?”, the system can determine that hemorrhoids is the issue that the user wants to know more about. The system will search for the most important nutrients related to this issue, such as vitamin B2, B6, or C, and then find the ingredients that are rich with these nutrients. Finally, it will recommend the retrieved recipes. Ting et al. [14] constructed the Dietary Recommendation System (DRS) with the use of personal data, such as height, weight, gender, lifestyle, and medical history, such as diabetes and high blood pressure, as well as cooking preferences.

Taking Taiwan as an example, the prevalence of obesity, diabetes, hypertension, and triglycerides is rapidly rising, which is related to Taiwanese people’s traditional lifestyle [15]. Specifically, the intake of excessive amounts of refined sugar [16]. Moreover, the 1993–1996 and 2005–2008 surveys of the Nutrition and Health Survey in Taiwan (NAHSIT) found that the most serious problem and the increasing prevalence among Taiwanese people are obesity and obesity-related metabolic abnormal diseases, especially high blood sugar and triglyceride [16,17]. The survey also showed that Taiwanese aged 19–64 consume insufficient fruit, dairy products, and nuts, while the intake of protein, cholesterol, and saturated fatty acids is too high. Among them, the age group of 19–30 years old had more problems with insufficient vegetables [16]. Besides, excessive sodium intake is also one of the dietary problems in Taiwan. Sodium intake plays a pivotal role in the control of blood pressure, while the hypertensive disease is exactly the eighth leading cause of death among Taiwanese people as indicated by the Ministry of Health and Welfare, Taiwan. Studies have found that high sodium intake in the diet is associated with increased blood pressure [18,19]. The 2004 report of the Joint National Committee (JIN7) recommended that the treatment of hypertension involves maintaining the ideal weight. The intake of more fruits, vegetables, high fiber food, and less refined cereals can lower the risk of metabolic syndrome [20]. Therefore, how to improve our health through a better diet is a critical issue that we intend to tackle using machine learning and data mining technologies.

Recent studies have focused on nutritional analysis of recipes uploaded by users to well-known recipe sharing websites. A cross-sectional study included the nutritional composition of 5237 recipes on the AllRecipe website. They examined substances such as protein, carbohydrate, sugar, sodium, fat, saturated fat and dietary fiber in the recipe. Using the World Health Organization (WHO) health score (0–7 points, 0 is very unhealthy, 7 is very healthy) and the Food Safety Light Mark (Green Light: Healthy, Yellow Light: Normal, Red Light: Unhealthy) of the Food Safety Authority of the United Kingdom as an indicator, they analyzed whether online recipes meet high health standards. They found that most recipes are high in protein, fat, saturated fat and sodium, and the dietary fiber content is low, which is less healthy than recipes from TV programs. In addition, when analyzing the user’s favorite recipes, it is also found that popular recipes are usually less healthy [21]. Said and Bellogín [22] explored the relevance of obesity prevalence rates across the US using 170,000 users’ recipe preferences, 540 million recipes which included 8400 ingredients. They were able to identify users with higher obesity risks. Sajadmanesh et al. [1] conducted a large-scale recipe study to learn about cooking and cooking habits around the world. The Yummly Recipe website was used to collect more than 200 different cuisines and more than 150,000 recipes. They analyzed the ingredients of different regional cuisines in order to increase diversity. Their results showed that nutrients that are positively associated with obesity and diabetes are carbohydrates and sugars, which may be affected by the main ingredients of cakes and whipped cream, resulting in higher medical expenses. Conversely, protein has a significant negative correlation with diseases such as obesity and diabetes. Moreover, Su et al. [23] examined the food.com (renamed as Genius Kitchen now) recipe website using 5037 ingredients, with an average of 8.57 ingredients per dish. They conducted cuisines classification by ingredients as eigenvalues. The results can be applied to the recommended food category labelling and automatic classification of recipes. Kusmierczyk and Nørvåg [24] analyzed the interaction data of recipes and scores uploaded by the German community platform Kochbar.de and found that the changes in nutrition (fat, protein, carbohydrate and calories) in the diet have obvious temporal trends. Rokicki et al. [25] studied the difference in nutritional value between recipes uploaded by different user groups. In addition, the carbohydrate amount in recipes seems to decrease as the user age increases.

Contrastively, only a few studies investigated the correlation between recipes and health conditions in Taiwan. Considering recent advances in machine learning technologies, we believe applying them to this topic can be fruitful. Therefore, this paper aims to discover the correlation between food and health by exploring cuisines and its unique ingredients, building cuisine classification models and clustering nutrients. We start by collecting information from the largest Chinese recipe website in Taiwan, iCook.com. It is a social platform for amateurs to share and discuss cooking recipes. However, they lack standardized nutrient lists. Therefore, we use natural language processing (NLP) techniques to process the data in order to establish a food-centric vocabulary and database. Subsequently, we construct machine learning models to automatically classify the recipes. This research also extracts unique ingredients for each cuisine type and the nutritional information of each recipe by linking the recipe ingredients to the nutrient database. We then establish a clustering model of the recipe based on the nutrient characteristics, and finally explore the relationship between diet and health. Our contributions include findings from online Chinese recipes, the ingredients and nutrients of various cuisines, an analysis of the characteristics of various cuisines and their nutritional value, and the correspondence between diet and diseases. This research can help improve awareness of the effect of what we eat on our body, as well as propose customized recipes or recommendation services to individual users.

## 2. Materials and Methods

**Figure 1:**
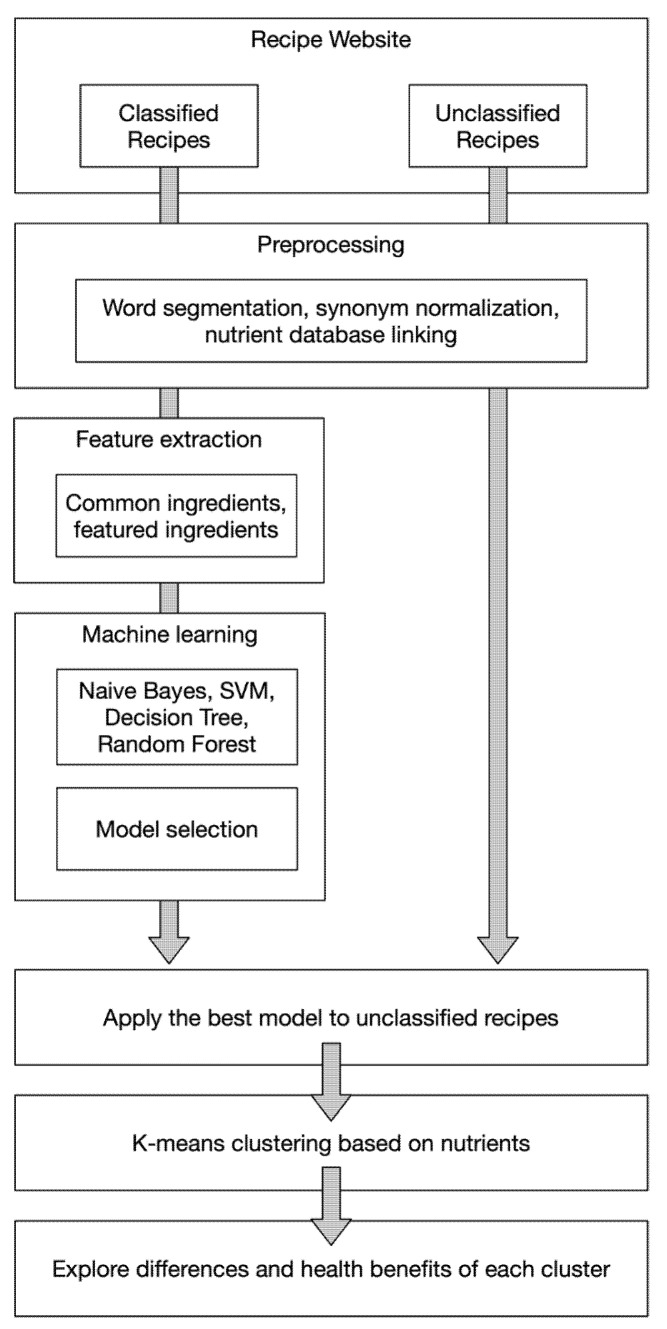
Framework of our analysis system.

Our study relies on the recipes data retrieved from iCook.tw, the largest Chinese recipe-sharing website in Taiwan. After data collection and preprocessing, ingredients and nutrients are regarded as features and machine learning as well as data exploration techniques are utilized to analyze cuisine types, dietary habits and its influences on health. The entire process of our system is presented in Figure 1. Major components of our method include: (1) preprocess the free-form recipes collected from the Internet, (2) train machine learning models to extract key features as well as help categorize these recipes by their nutritional content, and (3) uncover relationships between food, nutrients, and health. Detailed experimental methods are revealed in the following sections.

### 2.1. Data Collection

At the outset, we collect data from the recipe website including the most popular cuisines in Taiwan. Eight categories of cuisines are retained for further analysis, namely, Chinese (C), Japan (J), Korea (K), Thailand (T), America (A), Italy (I), France (F) and Spain (S) cuisines. The numbers of recipes in each cuisine category are 1321 (C), 1231 (J), 1333 (K), 1021 (T), 670 (A), 1836 (I), 949 (F), and 821 (S), thus resulting in 9182 classified recipes in total. In addition, there are also 6121 non-classified (N) recipes. This class of recipes are to be classified by the trained model later.

**Figure 2:**
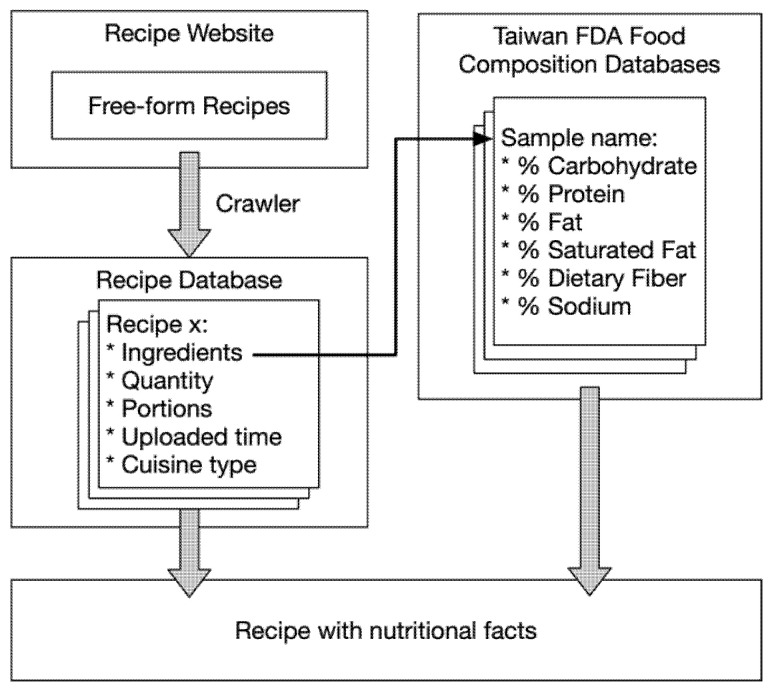
Linkage of online recipes and nutritional database to include rich nutrition information.

For the nutritional content, due to the fact that the website does not have nutritional labels, we attempt to map the ingredients to the 2017 version of the Taiwan FDA Food Composition Databases (TFDA) for gaining nutritional insights. This process is illustrated in Figure 2. The application of such a database can effectively convert free-form data into meaningful and structured information.

### 2.2. Data Preprocessing

The free-form data uploaded by users of the recipe website requires the following preprocessing steps. To start with, we need to normalize synonyms. It is common for the same ingredient to be written in different ways. Moreover, different physical forms of the same ingredient are commonly shown as different terms, for instance, “diced scallions” and “chopped scallions” both are scallions and should map to the same term in the nutrient database. Besides, we also remove parentheses, punctuations, and emojis, such as “「”, “【”, “】”, “！”, “
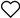
”, and all other non-ingredient-related words like kitchen appliances, cooking techniques such as “julienne.” Finally, in order to precisely analyze ingredients, the recipes that use “few, appropriate,” etc. quantity of the ingredients are replaced with an average amount. Decorative ingredients with a very small amount are removed. After preprocessing, we discover that there are some unrecognizable recipes or recipes with only one ingredient left. These exceptional cases are examined manually by the authors and excluded from the database.

### 2.3. Common Ingredients and Featured Ingredients

**Figure 3:**
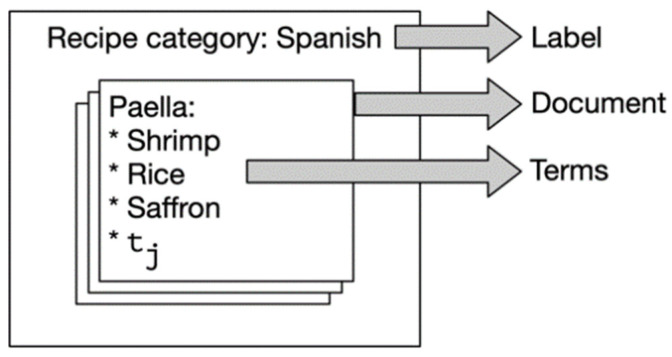
Different levels of a recipe formalized as their corresponding features.

This step is to identify common and featured ingredients, in other words, those that appear across multiple types and only in a few types. To achieve this, each recipe is considered as a document, while each ingredient $t_{j}$ is considered as a term and each cuisine type is considered as a label, as illustrated by Figure 3. Term Frequency—Inverse Document Frequency (tf-idf) (https://en.wikipedia.org/wiki/Tf%E2%80%93idf (accessed on 22 May 2021).) is used for calculating a vector representation of the ingredients. Then, we experiment with four widely used machine learning models, including naïve Bayes (NB) [26], decision tree (DT) [27], random forest (RF) [28], and support vector machine (SVM) [29], to construct our classifier.

More specifically, we calculate the importance and uniqueness of the ingredient terms in each recipe by using the tf-idf model, and then combine the above weights to obtain the “ingredient characteristic value”$\;p_{i,j}$ as:

$$p_{i,j}=\frac{r_{i,j}}{s_{j}}$$

The more times a certain ingredient appears in the recipe, the more important it is. Hence, we consider the frequency of an ingredient appearing in the cuisine type as a measure of its importance of the ingredient *i* in the category *j* cuisine as $p_{i,j}$. The denominator $s_{j}$ is the total number of recipes in cuisine type *j*. The numerator $r_{i,j}$ is the number of recipes in category *j* that includes ingredient *i*. At the experimental stage, a critical value $\tau_{i}$ is empirically chosen such that $p_{i,j}\geq \tau_{i}$ is the criterion for an ingredient to be considered as “featured” in the category *j*.

On the other hand, we define $w_{i,j}$ as a measure of the uniqueness of an ingredient *i* as the following equation. The numerator *N* is the total number of cooking categories, and the denominator $C_{i}$ is the number of cooking categories that include ingredient *i*.

$$w_{i,j}=\frac{N}{C_{i}}$$

Finally, by multiplying the importance score $p_{i,j}$ and the uniqueness weight $w_{i,j}$, we can obtain the “Specialty score” of the ingredient *i* in the category *j*, denoted as $S_{i,j}$. A higher Specialty score indicates that this ingredient has a higher chance of being a featured item in a certain category.

$$S_{i,j}=p_{i,j}*w_{i}$$

### 2.4. Nutrient Normalization

To understand the nutrition attributes of each cuisine, we extract nutritional information of nutrients by referring to the TFDA database mentioned in the previous section. We calculate nutritional facts per 100g of each recipe from each ingredient, and select seven of the most important and common nutrients, including carbohydrates, proteins, fat, saturated fat, dietary fiber, sugars and sodium. For an ingredient that cannot be mapped to the TFDA database, if the usage of it is low in the recipe, it is excluded in the calculation. Otherwise, it is replaced by the most similar ingredient in the TFDA database. Additionally, the quantity of some ingredients in the recipe is not specified using a precise unit, but usually described as “properly,” “a few,” etc. Therefore, we define the unspecified quantity of an ingredient by the following criteria:

i. Seasonings are usually set as the mean value.
ii. When the unit is described as “a long piece of,” “a piece of,” “a carton of,” etc., we use the food replacement table as a reference for the replacement of nutrients.
iii. The ingredients for decoration or spices with small amounts such as white sesame and mint leaf are excluded in calculation.

### 2.5. Cuisine Categorization

We utilize unsupervised learning (https://en.wikipedia.org/wiki/Unsupervised_learning (accessed on 22 May 2021).) methods to find groups that contain similar nutritional features withing the recipe. In other words, we do not use predefined category labels but rather their actual nutrients as the categorization criteria. In our experiments, we employ the k-means algorithm to distribute 13,323 recipes into 20 clusters, and use tf-idf to determine the most representative ingredients of each cluster. Through the steps mentioned in this section, we can quantitatively examine the correlation between recipe, its ingredients, and the well-being of our body.

## 3. Result

### 3.1. Feature Extraction

First, we perform basic preprocessing steps mentioned above, and the numbers of samples before and after preprocessing are listed in Table 1. Afterwards, 80% of the data are used for training and 20% for testing. The *k*-fold cross-validation scheme with k = 10 is adopted in our experiments. Multi-class classification models are used for classifying recipes into 8 categories, including Chinese (C), Japan (J), Korea (K), Thailand (T), America(A), Italy (I), France (F), and Spain (S) cuisines. In addition, due to the fact that only using accuracy as the metric for performance evaluation can be prone to bias, we include Macro-average F1-score as a comprehensive metric for multi-class models.

The tf-idf algorithm is then used to calculate the “Specialty Score” of ingredients. Recall that, the higher the Specialty Score, the more important the ingredient is for the cuisine type. The scores are listed in Table 2 and Table 3.

### 3.2. Classification Results

**Figure 4:**
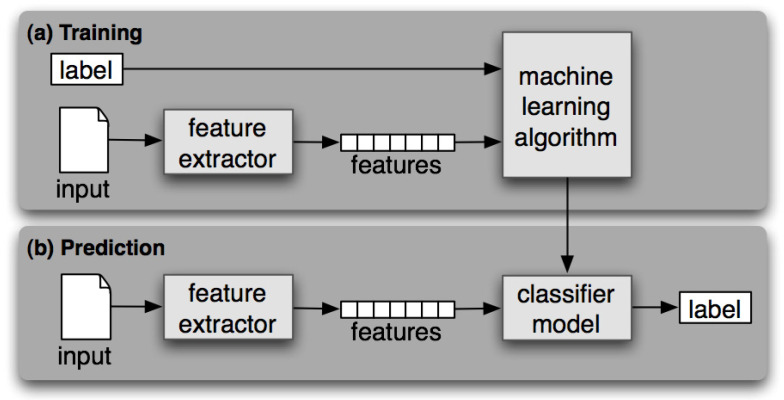
Classification model training and prediction process.

**Figure 5:**
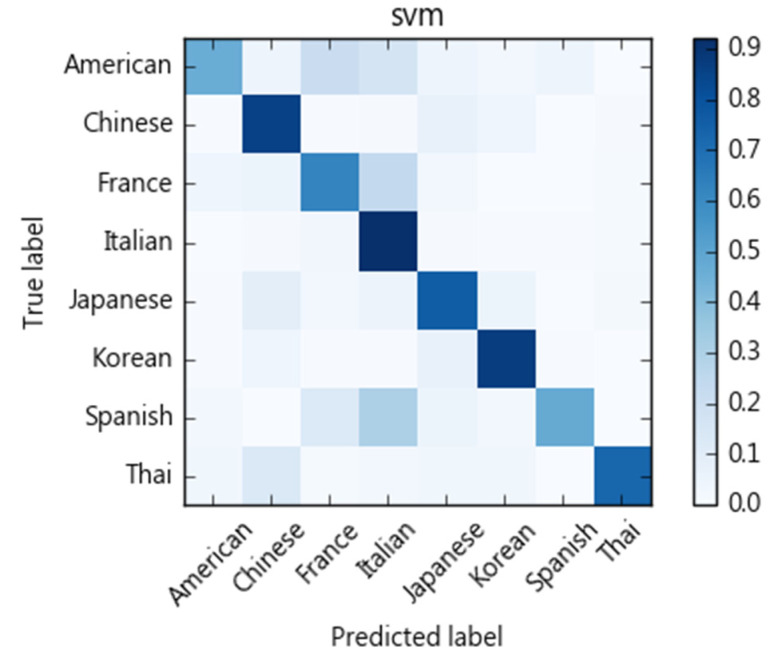
Confusion matrix of SVM classifier.

**Figure 6:**
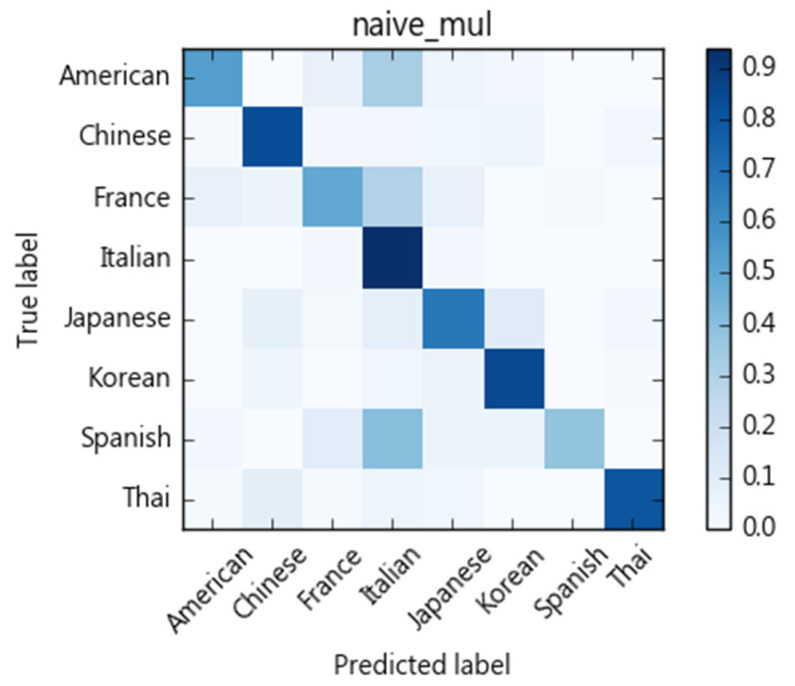
Confusion matrix of naïve Bayes classifier.

**Figure 7:**
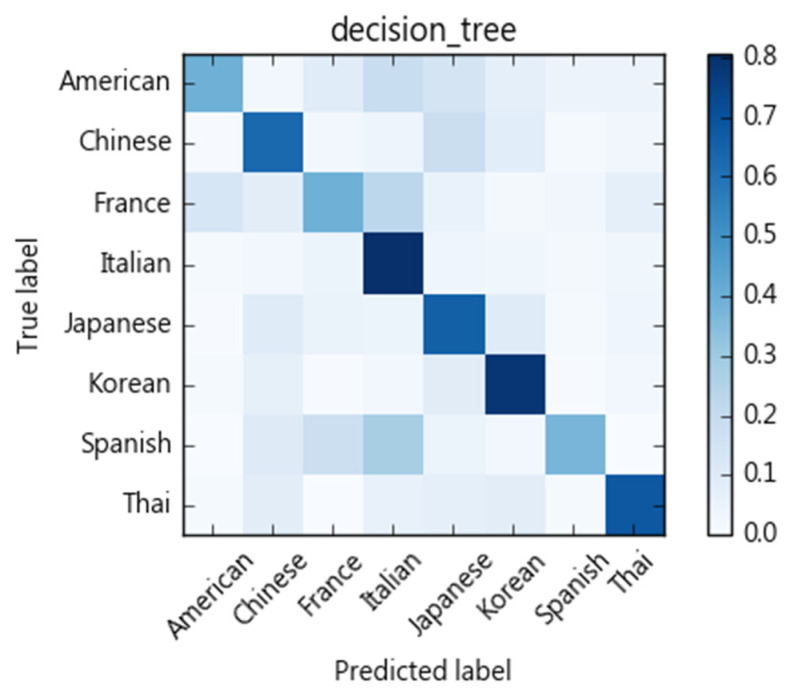
Confusion matrix of the Decision Tree classifier.

**Figure 8:**
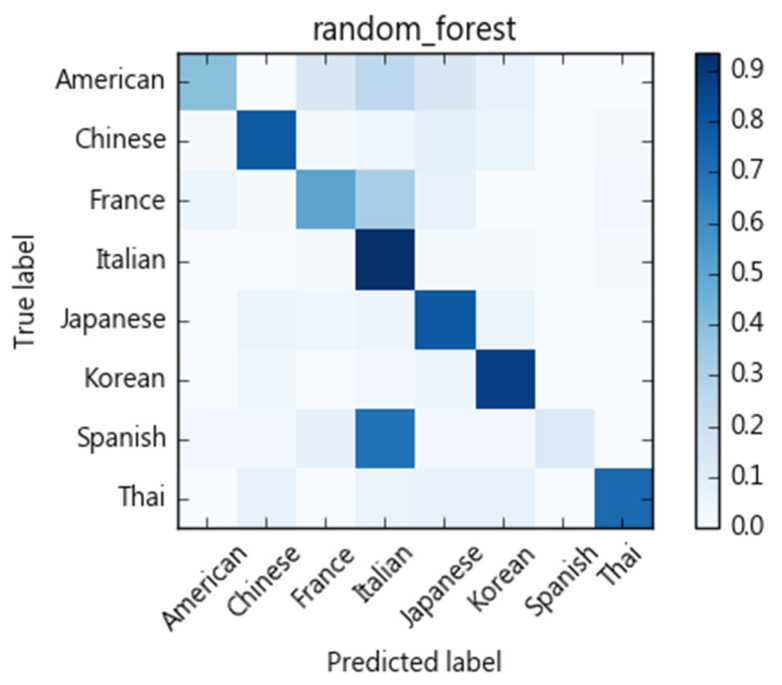
Confusion matrix of the random forest classifier.

We compare different classification models with regards to their ability to categorize recipes into eight cuisine types. As Figure 4 shows, each ingredient of a recipe is treated as a term. We then use tf-idf to calculate ingredient weights as a vector space feature representation. Here, cuisine types are treated as classification labels. We evaluate SVM, naïve Bayes, decision tree, and random forest for their classification performance. We adopt a 10-fold cross-validation scheme and calculate precision, recall, and F1-scores. The results of the SVM algorithm are shown in Table 4. The macro average of precision is 0.83, recall rate is 0.82, and F1-score 0.82. The confusion matrix is shown in Figure 5. Performances of compared methods are listed as follows: naïve Bayes model in Table 5 and its confusion matric in Figure 6, decision tree in Table 6 and Figure 7, and random forest in Table 7 and Figure 8.

**Figure 9:**
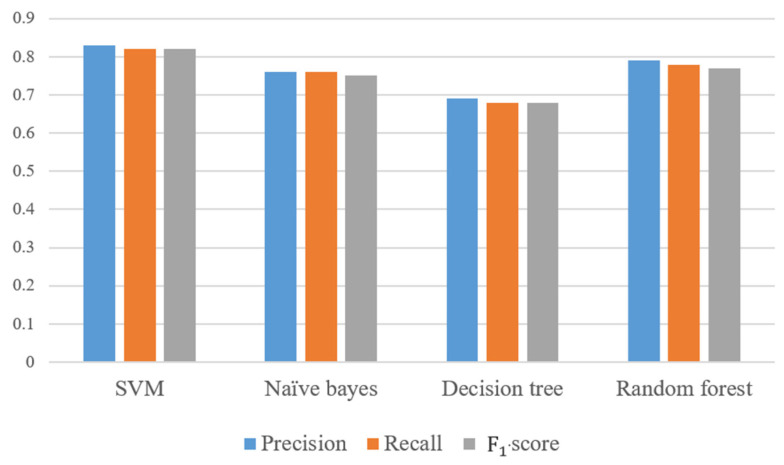
Comparison of classification models in terms of the macro-average precision, recall, and F1-score.

The macro average of three metrics from all classifiers are compared in Figure 9. Overall, we identify that the SVM model performs best on the categorization of recipes into cuisine types. Therefore, we adopt SVM to help us determine the category of the 5349 unclassified recipes collected from the web. The classification results are listed in Table 8.

### 3.3. Nutrient Grouping

The k-means algorithm is employed to distribute 13,323 recipes into 20 clusters. Afterwards, tf-idf is used to find representative ingredients of each cluster. We then manually label and merge them with similar characteristics. In the end, 20 clusters are reorganized into 5 groups, and results are as follows.

Group A: This group includes the 1st, 5th, and 13th clusters. The recipes in this group constitute a third of the total recipes. Carbohydrates, protein and fat are relatively average in this group (see Table 10 for a comparison). It also shows that the 13th cluster mainly uses low fat meat, such as salmon and chicken, as ingredients. Past researches indicate that high protein, low fat and low carbohydrates are good for weight loss [30]. Therefore, Group A is a healthier choice than Groups B, C, E, described later.

Group B: It contains the 2nd, 8th, 11th, and 19th clusters. This group has higher carbohydrates. Past studies have found that, under the same calorie limit, a diet that maintains a high carbohydrate ratio daily is not helpful to weight loss [31]. The 2nd cluster and the 8th also have a higher percentage of refined sugar. According to the World Health Organization, excessive amounts of sugar in food can cause obesity. In addition, many studies have indicated that excessive intake of sugar can increase triglycerides [32], total cholesterol [9], blood pressure [33], and cardiovascular disease [34]. It has a significant impact on our health. In the 2nd and 8th clusters, the recipes closest to the center of mass are mostly American recipes. The recipes mainly include cakes, muffins, crepes and other desserts.

Group C: It consists the 0th, 6th, and 12th clusters. This group has high sodium content, that is, high salt content. Many studies have shown that excessive sodium content in the diet has been considered to be associated with hypertension, cardiovascular disease, and chronic kidney disease [35,36,37]. Notably, the 6th and 12th clusters are mainly Japanese and Chinese dishes, respectively. Most of them require a cooking method of stewing or contain marinated ingredients. The representative ingredients in this group are soy sauce, rice wine, and chili.

Group D: It is made of the 4th, 10th, 14th, and 16th clusters. The main characteristic of this group is high dietary fiber. There is considerable epidemiological evidence that higher daily dietary fiber intake can reduce the risk of diseases including cardiovascular disease [38], Type 2 diabetes [17] and cancer [39]. Among the 17 recipes in this group, those closest to the center of mass are mostly Japanese, and those with high dietary fiber content are sushi. Some of the recipes with higher dietary fiber are listed in Table 9. Besides, most of them have a higher proportion of vegetables, or soybean-related products such as tofu, which are healthier food choices. 17 Japanese recipes and 9 Chinese recipes are closest to the center of mass. There are no Spanish cuisines and only one American recipe. Seaweed and onions are the representative ingredients of this group.

Group E: It includes the 3rd, 7th, 9th, 15th, 17th, and 18th clusters. This group has an overall higher portion of fat. We observe that the recipes in this group are mostly salads, which contains ingredients such as sesame oil, olive oil and coconut oil. Olive oil is rich in monounsaturated fatty acids, and several studies have shown that it can lower the chance of stroke for patients with cardiovascular diseases [40] and has an important role in reducing these diseases [41]. For the 7th and 15th clusters, 10 Chinese recipes are closest to the center of mass. For the 9th, 17th, and 18th clusters, 12 American cuisines are closest to the center of mass. In this group, in addition to common seasonings, the most representative ingredients of the 7th and 15th clusters are pork belly, olive oil and sesame oil; for the 17th, 9th and 18th clusters are milk, cream and unsalted cream. A large-scale longitudinal study pointed out that, using whole grain foods as the control, the risk of cardiovascular disease is lower when consuming unsaturated than saturated fat. The study also pointed out that if there is a high frequency of eating fine starch and saturated fat at the same time, there is a higher prevalence rate of cardiovascular disease [42]. The recipes in this group are mostly cakes, pastries, etc., with low amount of dietary fiber. Observing the ingredients of this group, we notice that most of them use refined carbohydrates such as flour, bread, etc.

To summarize, our model finds 20 clusters of recipes that may have various influences on human health. Table 10 is a list of groups and clusters that are more in line with principles of a healthy diet, accounting for 60% of all recipes. On the other hand, Table 11 shows other groups that do not conform with these principles. Note that the recipe database we have so far only covers a portion of the online recipes in one website. Overall, Japanese recipes and dishes are generally healthier. However, other factors such as the fact that there are fewer French and Spanish recipes, and cooking methods are not considered. Among those listed in Table 11, most are American and French desserts with refined sugar, saturated fat, and carbohydrates. Another major trait of this group is high sodium content.

## 4. Discussion

### 4.1. Common Ingredients and Featured Ingredients

To acquire a deeper insight, we analyze common ingredients and featured ingredients of various cuisine types. We observe that Chinese, Japanese, and Korean cuisines use similar ingredients, whereas American, Italian, French, and Spanish cuisines are comparable in the same manner. Thai food is more distinct, where common ingredients such as salt, sugar, and pepper are rare. The reason may be that Thailand is in Southeast Asia, where the preference of flavor is different from Northeast Asia. More precisely, it focuses on sour, spicy, and umami. Lemon, chili, and fish sauce are common ingredients in this region. It is also customary for people in this area to use fish sauce to replace salt and/or sugar, so there is a considerable difference in the use of ingredients from other cuisines.

When Sajadmanesh et al. [1] used tf-idf to extract distinctive ingredients, they found a strong relationship with culture, geographical location, and agriculture. It was also shown that Western European cuisine is more similar to North American dished, both relying heavily on dairy products, eggs and wheat-based products. Asian cuisines commonly use soy sauce, sesame oil, rice, and ginger [45]. Our analysis on the contents of online recipes is consistent with the results of previous work.

### 4.2. Cuisine Classification Model

**Figure 10:**
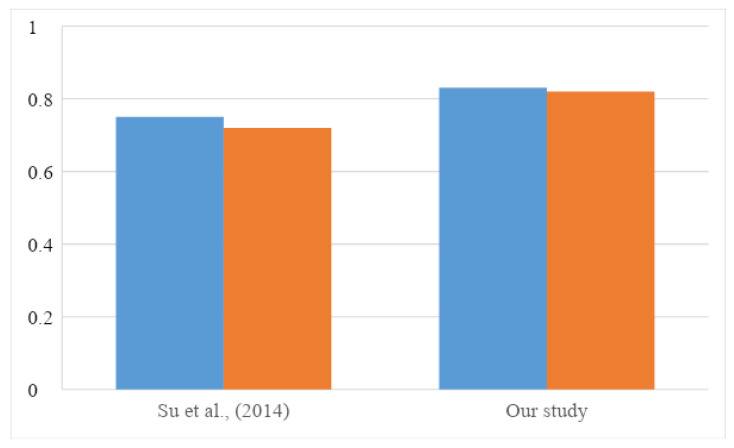
Comparison of model performances from Su et al. [23] and SVM model developed in this research.

In addition to the SVM classification model, the current study also uses naïve Bayes, decision tree, and random forest to compare the results. SVM was previously used by Su et al. [23] to understand the relationship between cuisines and ingredients based on the presence or absence of ingredients. Similar to our methods, their study used online recipes data for cuisine type prediction. We determine that, among all classification methods, the SVM model can obtain the best result. Furthermore, this study employs tf-idf to calculate the weight of each ingredient, and convert each recipe into a vector representation. Compared with sparse representation, the vector space model has the following advantages: (1) The weight of ingredients is not binary. (2) It can be sorted according to the degree of correlation between recipes. (3) It supports local matching. As shown in Figure 10, the SVM model in this research has a higher precision and recall than that from Su et al. [23].

Figure 5, Figure 6, Figure 7 and Figure 8 show the confusion matrices corresponding to SVM, naïve Bayes, decision tree, and random forest classifiers in this study. Compared to other cuisines, Chinese, Italian, Korean, Thai, and Japanese cuisines are easy to distinguish from one another, whereas French, American, and Spanish cuisines are more challenging. Taking the confusion matrix of the SVM model as an example, we can further find that American, French, and Spanish recipes tend to be classified as Italian cuisine. Table 12 lists some American, French, and Spanish recipes that are classified as Italian and their ingredients. We note that some ingredients have higher Specialty scores in Italian cuisine, but the misclassified recipes have low Specialty scores on those ingredients, which may be the reason for the misclassification of these dishes. For example, American recipes classified as Italian cuisine mostly contain olive oil, while French recipes include tomatoes, spaghetti and other ingredients, and Spanish recipes include basil, tomatoes, and cream. Similarly, Thai and Japanese dishes are sometimes classified as Chinese dishes. As indicated in Table 13, soy sauce, white pepper, rice wine, and shiitake mushrooms are also quite common ingredients in Chinese cuisine.

### 4.3. Relationship between Nutrients

**Figure 11:**
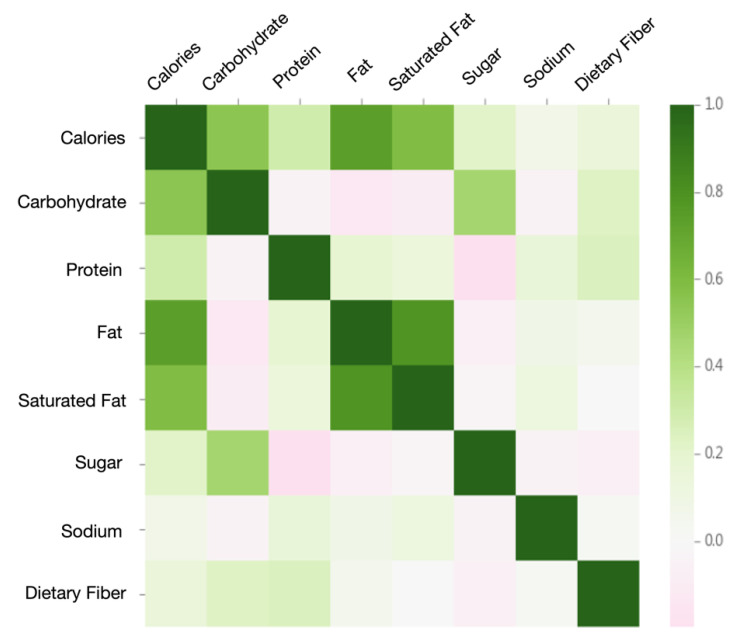
Pearson correlation matrix between nutrients in recipes.

Hsiao and Chang [46] stated that using recipe recommendations can greatly improve users’ dietary habits and health. Therefore, we further use the clustering results to explore the nutrient characteristics of recipes, and analyze whether each cluster of recipes is in line with principles of a healthy eating habit. For general ingredients, calories are provided by carbohydrates, proteins, and fats, where one gram can provide four calories, four calories and nine calories, respectively. Sodium and dietary fiber do not provide calories. Figure 11 shows the Pearson correlation coefficient matrix of seven nutrients, namely, carbohydrate, protein, fat, saturated fat, sugar, dietary fiber, and sodium, with calories. It can be perceived that all nutrients are positively correlated with calories. Interestingly, carbohydrates are negatively correlated with protein, fat, and saturated fat; and sugar has low correlations with fat, saturated fat, and sodium, while negatively correlated with protein. The result is consistent with past research [24]. It has been shown that dietary fiber is mostly found in whole wheat grains, vegetables, fruits, and soybeans. If more refined sugar is added to the recipe, such as granulated sugar, powdered sugar, etc., the recipes are mostly of exquisite pastries. Thus, the dietary fiber content is relatively low [3]. The findings in our experiments agree with previous research, in that dietary fiber is positively correlated with carbohydrates and protein, and negatively correlated with sugar.

For the purpose of suggesting a healthier diet, we refer to the Dietary Guidelines for Americans 2020–2025, Guideline 4 of Chapter 1 (https://www.dietaryguidelines.gov/sites/default/files/2020-12/Dietary_Guidelines_for_Americans_2020-2025.pdf (accessed on 22 May 2021)). It states that foods and beverages with added sugar, fat, sodium, or alcohol should be reduced. This corresponds to Groups B, C, and E in Section 3.3. These recipes are high in sugar, sodium, and fat content, which are related to obesity, high cholesterol, high blood pressure, heart disease, and chronic kidney disease. The Japanese Dietary Guidelines recommend moderate consumption of highly processed snacks, confectionery and sugar-sweetened beverages as well [47]. Furthermore, the Updated Mediterranean Diet Pyramid [48] also encourages healthy fats like olive oil to be the main source of fat, while sweets and ultra-processed high-sugar, high-fat foods and beverages should only be consumed in small amounts. These modernized views of healthy diets align well with Group E in Section 3.3. Moreover, the Italian Dietary Guidelines (http://www.fao.org/nutrition/education/food-dietary-guidelines/regions/countries/italy/en/ (accessed on 22 May 2021)) advocate for small amounts of fat and sugar in foods. To sum up, our method can successfully find groups of recipes and nutrients that are consistent with most up-to-date dietary recommendations around the world.

### 4.4. Strengths and Limitations

The strengths of our study include the use of state-of-the-art machine learning models to efficiently categorize recipes and discover relations among ingredients and nutrients, which enable an objective measure of healthy diets. Another advantage of our methods is its accuracy. A study of recipes provided by restaurants on university campuses [49] mentioned that the accuracy of menu labels has a significant impact on the nutritional information actually provided by the dishes. People who want to choose healthy meals may be affected by incorrect menu labels, which may in turn result in lower nutritional intake than expected, and even lead to problems with diet control. In our experiments, we use online recipes that provide detailed ingredients, and we also consult nutrition databases to achieve a complete nutritional analysis for each recipe. In this way, our analysis can provide users with accurate nutritional content analysis. However, the distribution of recipes from the data source may be imbalanced, and the ingredient to nutrition database is not always comprehensive, i.e., some ingredients are not recognized or found in the database. These factors can limit the outcome of our system.

## 5. Conclusions

This work examines ingredients and nutrients on a recipe sharing social network site iCook.tw to explore the health effects of various cuisines and ingredients. The online recipes are processed and nutrients within them are linked to a standard nutrient database. Multiple machine learning approaches are explored, and the SVM classifier is found to be superior in the recipe classification experiment than three other methods, with an F1-score of 0.82. We further analyze the healthiness of cuisines by clustering nutrients and organizing possible health effects of different clusters of recipes. We observe that only a third of the online recipes contain high protein, low fat and low carbohydrates, which are indications of a healthier diet. As for the most notable relationship between nutrients, sugar is negatively correlated with protein and dietary fiber, in other words, sweeter dishes are usually low in protein and fiber content. On the other hand, dietary fiber is positively correlated with carbohydrates and protein, which are essential nutrients of human health. Our findings can help the public to better understand the impact of dietary habits. We foresee more nutritious and healthy cooking styles to emerge after our proposal of the awareness of healthy cuisines and ingredients.

## Figures and Tables

**Table 1:** Number and conversion rate of recipes in each category before and after preprocessing.

Class	Chinese	Japanese	Korean	Thai	American	Italian	French	Spanish	Unknown	Total
**Before** **processing**	1321	1231	1333	1021	670	1836	949	821	6121	15,303
**After** **processing**	1211	1096	1212	920	562	1623	713	637	5349	13,323
**Conversion rate**	91.6%	89.0%	90.9%	90.1%	83.8%	88.3%	75.1%	77.5%	87.3%	87.6%

**Table 2:** Chinese, Japanese, Korean, and Thai cuisines’ Specialty score of ingredients.

Chinese		Japanese		Korean		Thai	
Ingredients	Specialty Score	Ingredients	Specialty Score	Ingredients	Specialty Score	Ingredients	Specialty Score
Soy sauce	0.44	Soy sauce	0.47	Onion	0.40	Fish sauce	0.55
White pepper	0.37	Seaweed	0.26	Soy sauce	0.34	Lemon	0.42
Chinese mushroom	0.28	Mirin	0.32	Kimchi	0.33	Coriander	0.28
Rice wine	0.27	Onion	0.26	Chili sauce	0.25	Chili	0.27
Shallot	0.21	Carrot	0.25	White sesame	0.25	Onion	0.26
Dried sea shrimp	0.20	Sushi	0.22	Carrot	0.24	Basil	0.18
Tapioca flour	0.19	Cabbage	0.18	Rice cake	0.22	Soy sauce	0.13
Sesame oil	0.18	Bonito flakes	0.17	Sesame oil	0.21	Sweet and chili sauce	0.12
Pork	0.16	Rice wine	0.13	Korean hot sauce	0.19	Lemongrass	0.11
Rice noodles	0.15	Cucumber	0.12	Chili powder	0.18	Coconut milk	0.10

**Table 3:** American, Italian, French, and Spanish cuisines’ Specialty score of ingredients.

American		Italian		French		Spanish	
Ingredients	Specialty Score	Ingredients	Specialty Score	Ingredients	Specialty Score	Ingredients	Specialty Score
Butter	0.37	Spaghetti	0.58	Milk	0.45	Onion	0.44
Milk	0.35	Onion	0.40	Butter	0.40	Black pepper	0.34
Black pepper	0.33	Black pepper	0.34	Black pepper	0.29	Olive oil	0.28
Chocolate	0.28	Olive oil	0.31	Onion	0.28	Saffron	0.27
Unsalted butter	0.25	Cheese	0.21	Unsalted butter	0.20	Sweet pepper	0.24
Low-gluten flour	0.21	Tomato	0.14	Low-gluten butter	0.19	Potato	0.23
Baking powder	0.19	Butter	0.13	Olive oil	0.18	Clam	0.20
Vanilla extract	0.18	Milk	0.11	Cheese	0.16	Lemon	0.19
Medium-gluten flour	0.17	Basil	0.11	Cream	0.15	Tomato	0.18
Ketchup	0.17	White wine	0.10	Lemon	0.12	White wine	0.17

**Table 4:** Performance of the SVM classifier for cuisine types.

Cuisine Types	Precision	Recall	F1-Score
American	0.71	0.52	0.60
Chinese	0.80	0.90	0.84
French	0.53	0.63	0.58
Italian	0.84	0.94	0.88
Japanese	0.81	0.78	0.80
Korean	0.91	0.83	0.87
Spanish	0.89	0.41	0.56
Thai	0.91	0.78	0.84
macro-average	0.83	0.82	0.82

**Table 5:** Performance of the naïve Bayes classifier.

Cuisine Types	Precision	Recall	F1-Score
American	0.56	0.55	0.55
Chinese	0.82	0.79	0.81
French	0.47	0.47	0.47
Italian	0.73	0.91	0.81
Japanese	0.75	0.69	0.72
Korean	0.80	0.78	0.79
Spanish	0.71	0.31	0.43
Thai	0.88	0.73	0.80
macro-average	0.76	0.76	0.75

**Table 6:** Performance of the Decision Tree classifier.

Cuisine Types	Precision	Recall	F1-Score
American	0.34	0.39	0.37
Chinese	0.76	0.63	0.69
French	0.42	0.44	0.43
Italian	0.75	0.81	0.78
Japanese	0.54	0.69	0.61
Korean	0.79	0.70	0.74
Spanish	0.58	0.36	0.44
Thai	0.72	0.67	0.69
macro-average	0.69	0.68	0.68

**Table 7:** Performance of random forest classifier.

Cuisine Types	Precision	Recall	F1-Score
American	0.59	0.39	0.47
Chinese	0.83	0.80	0.81
French	0.50	0.44	0.47
Italian	0.75	0.95	0.84
Japanese	0.75	0.81	0.78
Korean	0.88	0.84	0.86
Spanish	0.88	0.18	0.30
Thai	0.94	0.74	0.83
macro-average	0.79	0.79	0.78

**Table 8:** Distribution of category of the unclassified recipes as predicted by the SVM model.

Category	Chinese	Japanese	Korean	Thai	American	Italian	French	Spanish	Total
**Number**	1264	961	1272	584	315	571	382	264	5349

**Table 9:** Recipes in Group D that contains higher proportions of dietary fiber.

Category	Recipe	Ingredients
Japanese	Perfect Gunkanmak Sushi	Cucumber, grain rice, bell pepper, mayonnaise, goji berries, seaweed flakes
Japanese	Japanese style tofu	Tofu, mashed radish, bread flour, sweet potato flour, seaweed, bonito flakes, egg sauce, bonito sauce
Italian	Smoked salmon spaghetti	Beef tomato, spaghetti, onion, sea salt, olive oil, cheese powder, smoked salmon, fish, cauliflower, mushrooms, red onion

**Table 10:** Clusters and groups that conform with principles of healthy diets.

Group	Cluster	Nutrient Characteristics	Diet Habits and Disease Risk
A	1	balanced	Reduce the risk of obesity and metabolic diseases [43]
	5		
	13	balanced, protein	Good for weight loss [30]
B	2	carbohydrate	-
D	4	high dietary fiber	Reduce the risk of cardiovascular disease [38], Type 2 diabetes [17], cancer [39]
	10		
	14		
	16	carbohydrate	-
E	9	fat (provided by olive oil or coconut oil)	Reduce the risk of stroke [40] or cardiovascular disease [41]

**Table 11:** Clusters and groups that do not conform with principles of healthy diets.

Group	Cluster	Nutrient Characteristics	Diet Habits and Disease Risk
B	8	High carbohydrate	Not beneficial to weight loss [31]
	11	High sugar	Increase the risk of triglycerides [32], total cholesterol [44], blood pressure [33], cardiovascular disease [34]
	19		
C	0	High sodium	Related to hypertension [35], cardiovascular disease [36], chronic kidney disease [37]
	6		
	12		
E	15	High saturated fat, protein	Higher prevalence of cardiovascular disease [42]
	9	Highly saturated fat, carbohydrate and refined sugar	
	17		
	18		

**Table 12:** Examples of recipes that are misclassified as Italian.

Category	Recipes	Ingredients
American	Pan-fried steak + garlic bread	Olive oil *, steak, black pepper, cream, salt, garlic, lemon, bread
American	American Southwestern Grilled Chicken Leg	Chicken drumsticks, olive oil *, cream, spice powder
French	French wine stewed beef	Red wine, cream, beef brisket, bay leaf, tomato *, rosemary, dill, carrots, potatoes, onions
French	French Mushroom Pasta	Black pepper, mustard, shiitake mushrooms, white mushrooms, garlic, bacon, cheese, salt, whipped cream, pasta *, yogurt, onions
Spanish	Spanish stew with spicy meat sauce	Red pepper, basil *, black pepper, garlic, broth, bay leaf, tomato *, parsley, carrots, ground pork, onion
Spanish	Spanish Vegetable Baked Eggs	Milk*, cheese, spinach, tomato *, bell pepper, salt, egg, cream *, flour, onion

* Top ten featured ingredients of Italian cuisines, but the specialty value in this cuisine category is low.

**Table 13:** Examples of recipes that are misclassified as Chinese.

Category	Recipes	Ingredients
Thai	Stir fried pork with holy basil	Chili, garlic, nine-story pagoda, ground pork *, string beans, soy sauce *
Thai	Pandan leaf chicken	White pepper *, pandan leaves, shallots, rice wine *, chicken legs, ginger, lemongrass, salt, shallots, sesame oil *, white powder, soy sauce *
Japanese	Sukiyaki	Tofu, Shiitake mushroom *, pork belly, cabbage, egg, onion, Enoki mushroom
Japanese	Japanese fried chicken	Ginger, oil, garlic, cornmeal, chicken thigh, sugar, chestnut powder, white powder, white pepper *, egg, soy sauce, rice wine *

* Top ten featured ingredients of Chinese cuisines, but the specialty value in this cuisine category is low.

## References

[1] Sajadmanesh S., Jafarzadeh S., Ossia S.A., Rabiee H.R., Haddadi H., Mejova Y., Musolesi M., Cristofaro E.D., Stringhini G. Kissing Cuisines: Exploring Worldwide Culinary Habits on the Web. Proceedings of the 26th International Conference on World Wide Web Companion.

[2] Mejova Y., Haddadi H., Noulas A., Weber I. # foodporn: Obesity patterns in culinary interactions. Proceedings of the 5th International Conference on Digital Health.

[3] Trattner C., Rokicki M., Herder E. On the relations between cooking interests, hobbies and nutritional values of online recipes: Implications for health-aware recipe recommender systems. Proceedings of the 25th Conference on User Modeling, Adaptation and Personalization.

[4] Armstrong B., Doll R. (1975). Environmental factors and cancer incidence and mortality in different countries, with special reference to dietary practices. Int. J. Cancer.

[5] Keys A., Mienotti A., Karvonen M.J., Aravanis C., Blackburn H., Buzina R., Djordjevic B., Dontas A., Fidanza F., Keys M.H. (1986). The diet and 15-year death rate in the seven countries study. Am. J. Epidemiol..

[6] De Lorgeril M., Salen P., Martin J.-L., Monjaud I., Delaye J., Mamelle N. (1999). Mediterranean diet, traditional risk factors, and the rate of cardiovascular complications after myocardial infarction: Final report of the Lyon Diet Heart Study. Circulation.

[7] Kratz M., Baars T., Guyenet S. (2013). The relationship between high-fat dairy consumption and obesity, cardiovascular, and metabolic disease. Eur. J. Nutr..

[8] Folchetti L.D., Monfort-Pires M., de Barros C.R., Martini L.A., Ferreira S.R.G. (2014). Association of fruits and vegetables consumption and related-vitamins with inflammatory and oxidative stress markers in prediabetic individuals. Diabetol. Metab. Syndr..

[9] Hopping B., Erber E., Mead E., Sheehy T., Roache C., Sharma S. (2010). Socioeconomic indicators and frequency of traditional food, junk food, and fruit and vegetable consumption amongst Inuit adults in the Canadian Arctic. J. Hum. Nutr. Diet..

[10] Kendall C.W., Esfahani A., Jenkins D.J. (2010). The link between dietary fibre and human health. Food Hydrocoll..

[11] Melnik T.A., Spence M.M., Hosler A.S. (2006). Fat-related dietary behaviors of adult Puerto Ricans, with and without diabetes, in New York City. J. Am. Diet. Assoc..

[12] Aberg J. Dealing with Malnutrition: A Meal Planning System for Elderly. Proceedings of the AAAI Spring Symposium: Argumentation for Consumers of Healthcare.

[13] Mino Y., Kobayashi I. Recipe recommendation for a diet considering a user's schedule and the balance of nourishment. Proceedings of the 2009 IEEE International Conference on Intelligent Computing and Intelligent Systems.

[14] Ting Y.-H., Zhao Q., Chen R.-C. Dietary recommendation based on recipe ontology. Proceedings of the IEEE 6th International Conference on Awareness Science and Technology (iCAST).

[15] Ku P.-W., Fox K.R., McKenna J., Peng T.-L. (2006). Prevalence of leisure-time physical activity in Taiwanese adults: Results of four national surveys, 2000–2004. Prev. Med..

[16] Wu S.J., Pan W.H., Yeh N.H., Chang H.Y. (2011). Trends in nutrient and dietary intake among adults and the elderly: From NAHSIT 1993–1996 to 2005–2008. Asia Pac. J. Clin. Nutr..

[17] Ye E.Q., Chacko S.A., Chou E.L., Kugizaki M., Liu S.M. (2012). Greater Whole-Grain Intake Is Associated with Lower Risk of Type 2 Diabetes, Cardiovascular Disease, and Weight Gain. J. Nutr..

[18] Cutler J.A., Follmann D., Allender P.S. (1997). Randomized trials of sodium reduction: An overview. Am. J. Clin. Nutr..

[19] He F.J., Li J., MacGregor G.A. (2013). Effect of longer term modest salt reduction on blood pressure: Cochrane systematic review and meta-analysis of randomised trials. Bmj.

[20] Baxter A.J., Coyne T., McClintock C. (2006). Dietary patterns and metabolic syndrome—A review of epidemiologic evidence. Asia Pac. J. Clin. Nutr..

[21] Trattner C., Elsweiler D., Howard S. (2017). Estimating the Healthiness of Internet Recipes: A Cross-sectional Study. Front. Public Health.

[22] Said A., Bellogín A. You are What You Eat! Tracking Health Through Recipe Interactions. Proceedings of the 6th Workshop on Recommender Systems and the Social Web (RSWeb 2014).

[23] Su H., Lin T.-W., Li C.-T., Shan M.-K., Chang J. Automatic recipe cuisine classification by ingredients. Proceedings of the 2014 ACM International Joint Conference on Pervasive and Ubiquitous Computing: Adjunct Publication.

[24] Kusmierczyk T., Nørvåg K. Online food recipe title semantics: Combining nutrient facts and topics. Proceedings of the 25th ACM International on Conference on Information and Knowledge Management.

[25] Rokicki M., Herder E., Demidova E. What’s On My Plate: Towards Recommending Recipe Variations for Diabetes Patients. Proceedings of the UMAP Workshops.

[26] McCallum A., Nigam K. A comparison of event models for naive bayes text classification. Proceedings of the AAAI-98 Workshop on Learning for Text Categorization.

[27] Safavian S.R., Landgrebe D. (1991). A Survey of Decision Tree Classifier Methodology. IEEE Trans. Syst. Man Cybern..

[28] Breiman L. (1999). Random Forests.

[29] Hearst M.A., Dumais S.T., Osuna E., Platt J., Scholkopf B. (1998). Support vector machines. IEEE Intell. Syst. Their Appl..

[30] Halton T.L., Hu F.B. (2004). The effects of high protein diets on thermogenesis, satiety and weight loss: A critical review. J. Am. Coll. Nutr..

[31] Noakes M., Keogh J.B., Foster P.R., Clifton P.M. (2005). Effect of an energy-restricted, high-protein, low-fat diet relative to a conventional high-carbohydrate, low-fat diet on weight loss, body composition, nutritional status, and markers of cardiovascular health in obese women. Am. J. Clin. Nutr..

[32] Malik V.S., Hu F.B. (2015). Fructose and cardiometabolic health: What the evidence from sugar-sweetened beverages tells us. J. Am. Coll. Cardiol..

[33] Te Morenga L.A., Howatson A.J., Jones R.M., Mann J. (2014). Dietary sugars and cardiometabolic risk: Systematic review and meta-analyses of randomized controlled trials of the effects on blood pressure and lipids. Am. J. Clin. Nutr..

[34] Maersk M., Belza A., Stødkilde-Jørgensen H., Ringgaard S., Chabanova E., Thomsen H., Pedersen S.B., Astrup A., Richelsen B. (2012). Sucrose-sweetened beverages increase fat storage in the liver, muscle, and visceral fat depot: A 6-mo randomized intervention study. Am. J. Clin. Nutr..

[35] Blackwood A.M., Sagnella G.A., Cook D.G., Cappuccio F.P. (2001). Urinary calcium excretion, sodium intake and blood pressure in a multi-ethnic population: Results of the Wandsworth Heart and Stroke Study. J. Hum. Hypertens.

[36] Polonia J., Maldonado J., Ramos R., Bertoquini S., Duro M., Almeida C., Ferreira J., Barbosa L., Silva J.A., Martins L. (2006). Estimation of salt intake by urinary sodium excretion in a Portuguese adult population and its relationship to arterial stiffness. Rev. Port. Cardiol..

[37] Thijssen S., Kitzler T.M., Levin N.W. (2008). Salt: Its role in chronic kidney disease. J. Ren. Nutr..

[38] Threapleton D.E., Greenwood D.C., Evans C.E., Cleghorn C.L., Nykjaer C., Woodhead C., Cade J.E., Gale C.P., Burley V.J. (2013). Dietary fibre intake and risk of cardiovascular disease: Systematic review and meta-analysis. BMJ.

[39] Zeng H.W., Lazarova D.L., Bordonaro M. (2014). Mechanisms linking dietary fiber, gut microbiota and colon cancer prevention. World J. Gastrointest. Oncol..

[40] Samieri C., Feart C., Proust-Lima C., Peuchant E., Tzourio C., Stapf C., Berr C., Barberger-Gateau P. (2011). Olive oil consumption, plasma oleic acid, and stroke incidence: The Three-City Study. Neurology.

[41] Estruch R., Ros E., Martinez-Gonzalez M.A. (2013). Mediterranean diet for primary prevention of cardiovascular disease. N. Engl. J. Med..

[42] Li Y., Hruby A., Bernstein A.M., Ley S.H., Wang D.D., Chiuve S.E., Sampson L., Rexrode K.M., Rimm E.B., Willett W.C. (2015). Saturated Fats Compared With Unsaturated Fats and Sources of Carbohydrates in Relation to Risk of Coronary Heart Disease: A Prospective Cohort Study. J. Am. Coll. Cardiol..

[43] Simpson S.J., Le Couteur D.G., Raubenheimer D. (2015). Putting the Balance Back in Diet. Cell.

[44] Stanhope K.L., Medici V., Bremer A.A., Lee V., Lam H.D., Nunez M.V., Chen G.X., Keim N.L., Havel P.J. (2015). A dose-response study of consuming high-fructose corn syrup-sweetened beverages on lipid/lipoprotein risk factors for cardiovascular disease in young adults. Am. J. Clin. Nutr..

[45] Ahn Y.-Y., Ahnert S.E., Bagrow J.P., Barabási A.-L. (2011). Flavor network and the principles of food pairing. Sci. Rep..

[46] Hsiao J.-H., Chang H. SmartDiet: A personal diet consultant for healthy meal planning. Proceedings of the 2010 IEEE 23rd International Symposium on Computer-Based Medical Systems (CBMS).

[47] Fernandez M.L., Raheem D., Ramos F., Carrascosa C., Saraiva A., Raposo A. (2021). Highlights of Current Dietary Guidelines in Five Continents. Int. J. Environ. Res. Public Health.

[48] Serra-Majem L., Tomaino L., Dernini S., Berry E.M., Lairon D., Ngo de la Cruz J., Bach-Faig A., Donini L.M., Medina F.-X., Belahsen R. (2020). Updating the Mediterranean Diet Pyramid towards Sustainability: Focus on Environmental Concerns. Int. J. Environ. Res. Public Health.

[49] Feldman C., Murray D., Chavarria S., Zhao H. (2015). Menu label accuracy at a university's food services. An exploratory recipe nutrition analysis. Appetite.

